# Facile Synthesis of Visible Light-Induced g-C_3_N_4_/Rectorite Composite for Efficient Photodegradation of Ciprofloxacin

**DOI:** 10.3390/ma11122452

**Published:** 2018-12-03

**Authors:** Zhiming Sun, Xiangwei Zhang, Rui Zhu, Xiongbo Dong, Jie Xu, Bin Wang

**Affiliations:** 1 School of Chemical and Environmental Engineering, China University of Mining and Technology (Beijing), Beijing 100083, China; zhangxiangwei0613@126.com (X.Z.); 13121969011@163.com (R.Z.); 18810672030@163.com (X.D.); 13126852095@163.com (J.X.); 2 Beijing Key Laboratory of Clothing Materials R&D and Assessment, Beijing Engineering Research Center of Textile Nanofiber, School of Materials Science and Engineering, Beijing 100029, China

**Keywords:** rectorite, g-C_3_N_4_, photocatalysis, ciprofloxacin, wastewater treatment

## Abstract

A novel kind of g-C_3_N_4_/rectorite composite with high visible-light photoactivity was developed via a mild and cost effective two-step process. Ciprofloxacin (CIP), a typical antibiotic, was applied to evaluate the photoactivity of the received catalysts. Furthermore, the by-products of CIP photodegradation were analyzed and the possible degradation pathways were also discussed. Compared with bare photocatalysts, the received composite possessed well reusability and higher photoactivity towards CIP. According to the characterization analysis results, layered g-C_3_N_4_ was successfully immobilized on layered rectorite, which could not only promote its adsorption capacity but also provide more reactive sites for CIP adsorption and photodegradation. Compared with bare g-C_3_N_4_, the photoactivity of the prepared composite was significantly enhanced. The enhancement should be mainly due to the lower recombination rate of photogenerated carriers and the improved adsorption capacity toward CIP. This study demonstrated that the obtained g-C_3_N_4_/rectorite composite should be a promising alternative material in wastewater treatment.

## 1. Introduction

With the fast development of industry, a large volume of wastewater, particularly, the residuals of medicine such as ciprofloxacin (CIP) are excreted into the ground waters, which seriously threaten the existence and long-term development of human society due to their biotoxicity and refractory [1]. To address these issues, traditional solutions such as ultrafiltration [2], absorption [3,4] and biological treatment [5] have been widely applied. However, these traditional methods could not effectively eliminate and completely mineralize the CIP. In recent years, many researches have been investigated to explore an environmentally friendly and renewable technology to degrade these medicines. Among these reported technologies, photocatalysis has been proven to be a green and efficient route to resolve the environmental problems on account of its low production cost and easy-to-operate. Particularly, TiO_2_ as a photocatalytic material has gained considerable interests, which exhibits enormous application prospect in photocatalysis [6]. However, the practical application of the bare anatase TiO_2_ nanoparticles was limited to a certain extent due to their relatively higher band gap (around 3.2 eV). Compared with TiO_2_ nanoparticles, the band gap of g-C_3_N_4_ is lower (around 2.7 eV), leading to its preferable visible light-induced photoactivity in water splitting and removal of various contaminants [7]. Additionally, g-C_3_N_4_ could be simply synthesized by heating some nitrogen containing compounds, for example cyanamide [8], dicyandiamide [9], trithiocyanuric acid [10], melamine [11], triazine [12], heptazine derivatives [13], urea [14] and thiourea [15], which means that the preparation methods of g-C_3_N_4_ would be easy to operate and inexpensive. However, g-C_3_N_4_ photocatalyst would easily agglomerate in the process of thermal polycondensation, leading to the decrease in the active sites of the adsorption towards pollutant.

To solve these application problems of g-C_3_N_4_, a great number of methods have been reported, such as exfoliating the bulk g-C_3_N_4_ by using organic solvents, acid media, or base media [16]. Due to their high-cost and environmentally unfriendly defects, these above methods could not be widely used in practical application. In recent years, some natural minerals with unique lamellar structure, such as kaolinite [17], montmorillonite [18], illite [19] and so forth, have been widely used as the g-C_3_N_4_ carriers to hinder the agglomeration of g-C_3_N_4_ during the thermal polycondensation process. Among these natural layered minerals, the rectorite seems to be an attractive option as catalyst carrier to synthesized 2D/2D hierarchical structure. On one hand, rectorite has a regular interstratified structure consist of 1:1 ratio of a mica component and a montmorillonite component, so the properties of rectorite were similar to both mica and montmorillonite. On the other hand, it has relatively higher thermal stability, compared with the other natural layered minerals, which would maintain the structure stability during the thermal polycondensation process of g-C_3_N_4_ [20,21].

Hence, in our present study, a novel g-C_3_N_4_/rectorite composite was developed via a facile and cost-effective route. Ciprofloxacin (CIP) was selected as the typical contaminant to evaluate the photoactivity of samples. The possible degradation pathway was illustrated based on the ESI-MS results. In addition, the possible photodegradation mechanism was discussed based on the scavenger experiment results as well. This paper would provide new insight for natural layered mineral based composite photocatalysts and a fundamental understanding of g-C_3_N_4_/rectorite composite to degrade wastewater.

## 2. Materials and Methods

### 2.1. Materials

The rectorite (RE) used in the study was obtained from Zhongxiang city, Hubei province, China, which was used as the carrier of g-C_3_N_4_. Deionized water was applied to the whole experiment. Dicyandiamide (C_2_H_4_N_4_), ciprofloxacin (CIP) and other chemicals were purchased from Beijing Reagent Co. (Beijing, China). All chemicals used in this study were analytical grade without any further purification.

### 2.2. Catalysts Preparation

The bare g-C_3_N_4_ was obtained from a typical thermal polycondensation process of dicyandiamide [22]. Typically, 4.0 g dicyandiamide was put into an alumina crucible with a cover and heated to 550 °C in open air for 4 h at a heating rate of 2.3 °C·min^−1^. The resultant yellow product was collected for use without any further treatment. As a reference, the rectorite was also calcined at 550 °C (RE-550 °C) as same as the synthesis of bare g-C_3_N_4_ catalyst.

The g-C_3_N_4_/rectorite catalyst was prepared by a facile two-step process consisted of wet-chemical and calcination processes. In a typical synthesis, different qualities (1.0 g, 2.0 g, 3.0 g and 4.0 g) of dicyandiamide were dispersed into 60 mL of deionized water at 60 °C, while the solution was stirred continually. Afterwards, 1.0 g of rectorite was dissolved into above solution and stirred continually for 12 h. The resulted suspension was then treated at 105 °C for 10 h. Finally, the product was treated under the same thermal conditions as bare g-C_3_N_4_. The composites were designated as CNRE-1:1, CNRE-1:2, CNRE-1:3 and CNRE-1:4, respectively. According to the thermogravimetric analysis (TG), the mass ratio of g-C_3_N_4_ was measured as 65.4% in CNRE-1:3.

### 2.3. Characterization

The crystalline phase and structure were studied by X-ray powder diffraction (XRD) (Bruker, Karlsruhe, Germany) with Cu-Kα radiation (λ = 0.154056 nm). The surface character and morphology of samples were investigated by the S-4800 scanning electron microscopy (SEM) (Hitachi, Tokyo, Japan) equipped with an energy dispersive spectrum analysis (EDS). Photoluminescence (PL) spectra of as-prepared photocatalysts were received through the use of a fluorescence spectrophotometer (F-7000 PL) (Hitachi, Tokyo, Japan) with an emission wavelength of 360 nm. Fourier transformed infrared (FTIR) spectra was performed on a Nicolet iS10 spectrometer (Thermo Fisher Scientific, Waltham, MA, America) in the frequency range of 4000 and 600 cm^−1^. The reflectance spectra of as-prepared photocatalysts with the scope from 200nm to 800 nm were measured by a UV-vis spectrophotometer (UV-9000s, Shanghai Metash Instruments Co., Shanghai, China), which equipped with the diffuse reflectance accessory (DRS). The BET surface area and pore size distribution was assessed by JW-BK nitrogen adsorption-desorption isotherm analyzer apparatus (JWGB Sci. &Tech, Beijing, China) at 77 K.

### 2.4. Evaluation of Photocatalytic Activity

The photodegradation of CIP was carried out to measure the photoactivity. The visible light system was consisted of a 500 W Xenon lamp (BL-GHX-V, Shanghai Bilang plant, Shanghai, China) with a 420 nm cut-off filter to prevent the UV light from passing through. In the degradation experiment, 50 mg of sample (g-C_3_N_4_, CNRE-1:1, CNRE-1:2, CNRE-1:3 and RE) was added into the CIP solution (50 mL, 20 mg/L). As an object of comparative experiment, a physical mixture of rectorite and g-C_3_N_4_ was used to evaluate the photoactivity as well. The content of g-C_3_N_4_ in mixture was set as 65.4% (CNRE-MIX), same as CNRE-1:3. Subsequently, the ultrasonic vibration was used to make the suspension more uniformly. Then the mixture was transferred to the cylindrical reactor. Before the visible light illumination, the mixture was stirred without visible light for 30 min and then the catalysts and CIP would establish an adsorption/desorption equilibrium. At regular intervals, 4 mL of mixture was removed from the cylindrical reactor and centrifuged at 8000 rpm to separate pollutant and photocatalyst. An UV-vis spectrophotometer (UV-9000S, Shanghai Metash, Shanghai, China) was applied to monitor the removal performance for CIP. The maximum absorption of CIP was measured at 278 nm. Using the following formula, the degradation rate of CIP (D_R_) was evaluated:

D_R_ = (C_0_ − C_t_)/C_0_ × 100% (1)

where, C_0_ represents the concentration of CIP at initial time and C_t_ represents the concentration at time t. All the experiments were repeated for two times.

### 2.5. Analysis of Degradation by-Products of CIP

The photocatalytic mechanism was proposed by investigating the decomposition pathway of CIP. The possible intermediates from CIP degradation were determined using electrospray ionization mass spectrometry (ESI-MS, micrOTOF-Q II, Bruker, Karlsruhe, Germany). Full-scan spectra were measured by *m*/*z* scanning from 50 to 1000. The capillary voltage was set at 3000 V. The collision cell RF was set at 110.0 Vpp. The pressure of the nebulizer was set at 0.4 bar. The temperature of the dry heater was set at 180 °C. The flow rate of the dry gas was controlled at 2.0 L min^−1^.

## 3. Results and Discussion

### 3.1. XRD Analysis

**Figure 1:**
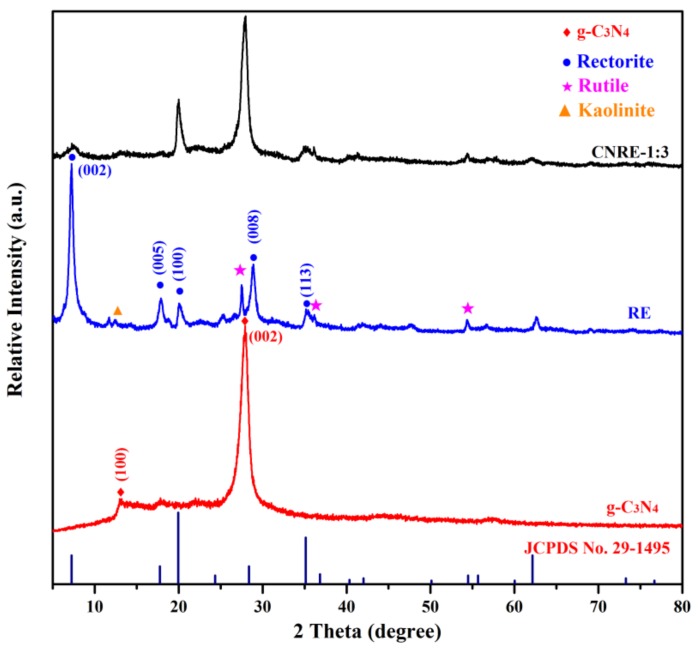
XRD patterns of g-C_3_N_4_, rectorite and CNRE-1:3.

The crystallographic and structural characteristics of rectorite, g-C_3_N_4_ and CNRE-1:3 were present in Figure 1. It is clear that rectorite displays two relative strong peaks at 7.25° and 28.89°, which could be indexed as the (002) and (008) lattice plane, respectively. This is well matched with the patterns of rectorite (JCPDS No. 29-1495) [23,24]. Other several peaks located at 17.88°, 20.04° and 35.40° are assigned to (005), (100) and (113) lattice planes, respectively. The peak at 12.47° represented the existence of the kaolinite in this sample [25]. In addition, the typical diffraction peaks of impurity rutile at 27.49°, 36.14°, 54.40°, 62.61° were also observed in rectorite [26]. Two peaks located at 13.08° and 27.58° are observed in g-C_3_N_4_, which are the characteristic peaks of g-C_3_N_4_. The stacking of g-C_3_N_4_ layers attribute to the diffraction peak at 27.58° (002) and diffraction peak at 13.08° (100), which could be assigned to the period structural of tristriazine units [22]. Simultaneously, these two diffraction peaks also appeared in the XRD pattern of CNRE-1:3, demonstrating that g-C_3_N_4_ was successfully immobilized on rectorite.

### 3.2. Microstructure Analysis

**Figure 2:**
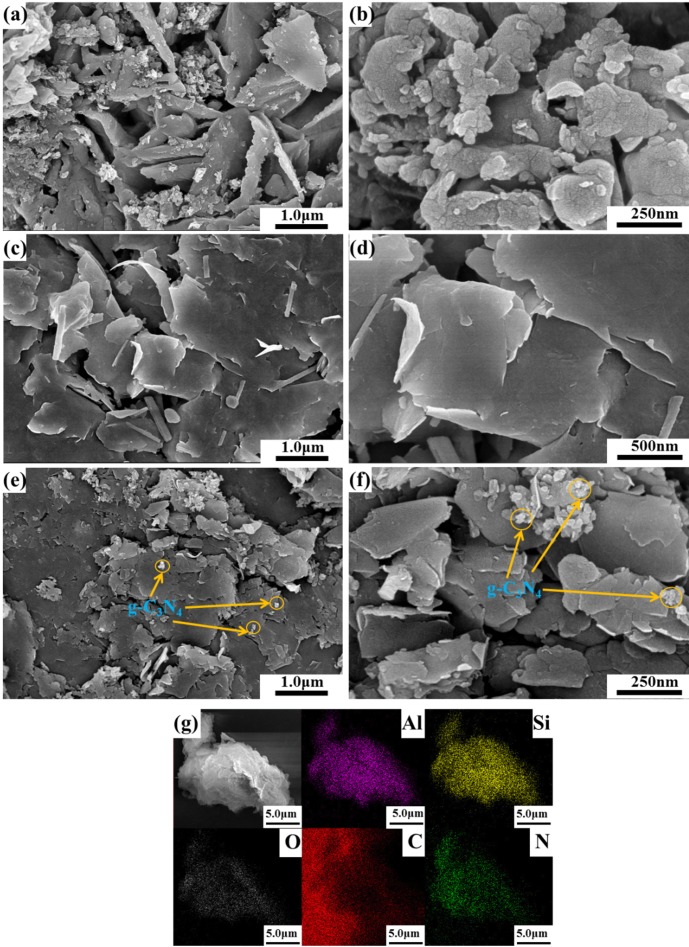
(**a**,**b**) SEM images of g-C_3_N_4_; (**c**,**d**) rectorite; (**e**,**f**) CNRE-1:3; (**g**) element mapping of Al, Si, O, C, N for CNRE-1:3 composite.

The microstructures of g-C_3_N_4_, rectorite and CNRE-1:3 were investigated with SEM analysis. As displayed in Figure 2a,b, the bare g-C_3_N_4_ possessed a typical aggregated layered structure, which should be attributed to the thermal polymerization of dicyandiamide. As seen in Figure 2c,d, the rectorite showed a layered structure composed of parallel nanosheets with a smooth surface. This lamellar structure would benefit for 2D g-C_3_N_4_ immobilization. Compared with the pure rectorite, the rougher surface of CNRE-1:3 (Figure 2e,f) could also prove the successfully immobilization of g-C_3_N_4_ nanosheets on the surface of rectorite. The elemental compositions of CNRE-1:3 were further detected by element mapping in element mapping mode. As can be seen from Figure 2g, it is indicated that the C, N, O, Al, Si are observed in composite and evenly distributed, which demonstrates the well distributions of g-C_3_N_4_ on layered rectorite. More importantly, the well distribution of these elements revealed that the coexistence and contact of g-C_3_N_4_ and rectorite.

### 3.3. Photoluminescence Analysis

**Figure 3:**
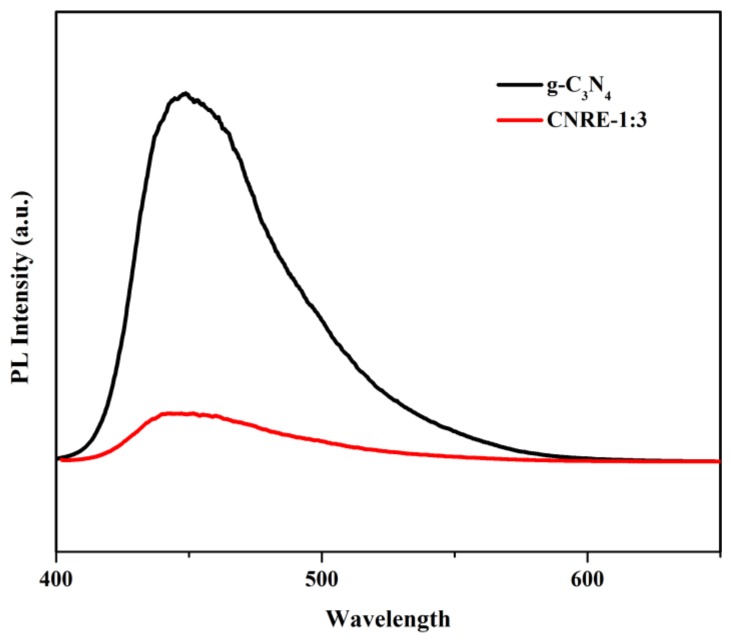
PL emission spectra (λex = 360 nm) of g-C_3_N_4_ and CNRE-1:3 composite.

The photoluminescence (PL) analysis was generally used to demonstrate the separation and recombination of electron-hole pairs by determining the charge-carrier trapping, transfer capacity of semiconductor under illumination [27]. The typical PL plots of g-C_3_N_4_ and CNRE-1:3 are observed in Figure 3. It is evidence that g-C_3_N_4_ displayed the similar peak position with that of CNRE-1:3 composite, observing the maximum peaks at around 450 nm because of band edge and defect emission [28]. However, compared to bare g-C_3_N_4_, the peak intensity of CNRE-1:3 was significantly decreased due to the introduction of rectorite. Generally, the sample with lower PL intensity stands for the higher separation efficiency of electron-hole pairs [29]. Hence, it is concluded that the immobilization of g-C_3_N_4_ nanosheets on the surface of rectorite could efficiently suppress the recombination of electron-hole pairs, enhancing the charge transfer in the photocatalytic process.

### 3.4. FTIR Analysis

**Figure 4:**
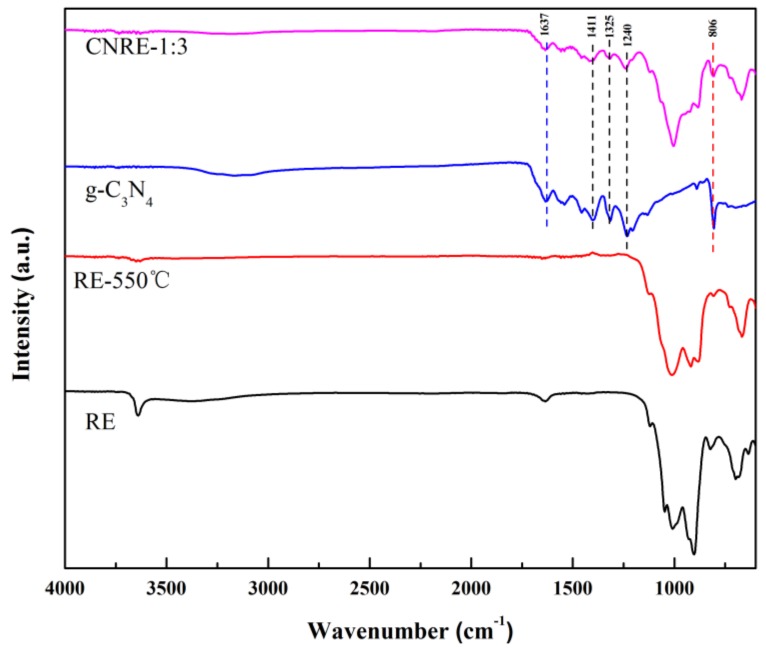
FTIR spectra of CNRE-1:3, g-C_3_N_4_, RE-550 °C and rectorite.

FTIR spectra of CNRE-1:3, g-C_3_N_4_, RE-550 °C and RE were displayed in Figure 4. The g-C_3_N_4_ revealed three main absorption regions. The peak at 3000–3500 cm^−1^ was attributed to the stretching vibration of N–H and surface adsorbed water molecules. The peak at 1637 cm^−1^ was due to the presence of C–N bonds and the peaks at 1411 cm^−1^, 1325 cm^−1^ and 1240 cm^−1^ were assigned to the typical vibration of aromatic rings [30,31]. The absorption peak at 807 cm^−1^ accorded with the characteristic breathing mode of triazine units [32]. For the rectorite, major peaks were in the range of 960–1150 cm^−1^, which could be ascribed to the Si–O stretching vibration of silica tetrahedron. The peak at 1635 cm^−1^ corresponded to the bending vibration of H_2_O. All the main characteristic vibration peaks of g-C_3_N_4_ and rectorite could be clearly found in the FTIR spectra of CNRE-1:3, suggesting the structure of g-C_3_N_4_ and rectorite remains intact after wet-chemical and calcination processes. However, no newborn functional groups were observed from the FTIR spectra analysis.

### 3.5. BET Analysis

**Figure 5:**
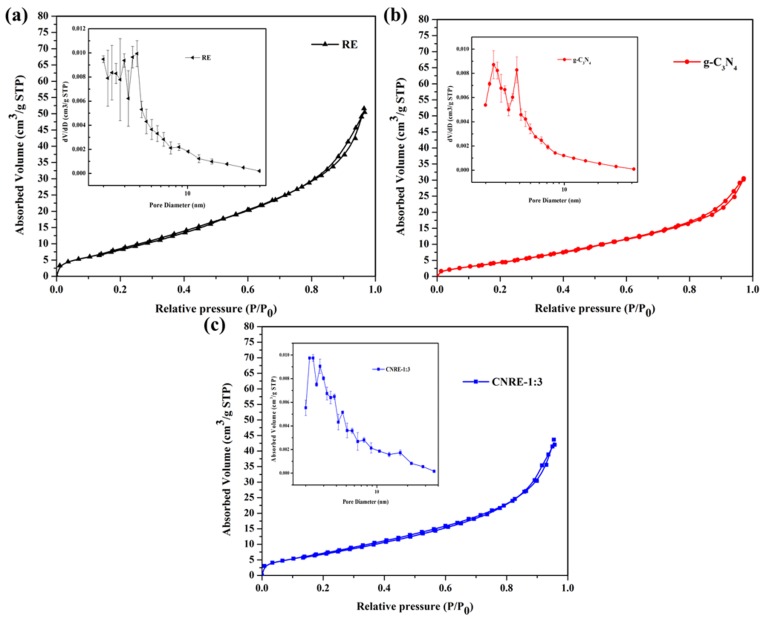
N_2_ adsorption-desorption isotherms measured at 77 K as well as BJH pore size distribution plots of (**a**) rectorite, (**b**) g-C_3_N_4_ and (**c**) CNRE-1:3.

The surface area and pore structure of RE, g-C_3_N_4_ and CNRE-1:3 were investigated by N_2_ adsorption-desorption isotherms and Barrett–Joyner–Halenda (BJH) pore size distribution plots. From Figure 5, the nitrogen adsorption-desorption isotherms plots of all samples exhibited a type II isotherm and the hysteresis loops were the type of H3, which typically present the formation of mesoporous structure with the pore size of 2–50 nm. As shown in Figure 5a,c, the pore size of CNRE-1:3 was similar with the RE, which demonstrated the introduction of g-C_3_N_4_ had almost no influence on the porous structure of rectorite. As presented in Table 1, the surface area of CNRE-1:3 and rectorite had almost the same surface area. It could be inferred that the structure of rectorite might not be destroyed during the calcination process at 550 °C. However, the surface area and pore volume of CNRE-1:3 were larger than those of g-C_3_N_4_, which would be beneficial for generating more adsorption and reactive sites for pollutant degradation.

### 3.6. Photocatalytic Activity

**Figure 6:**
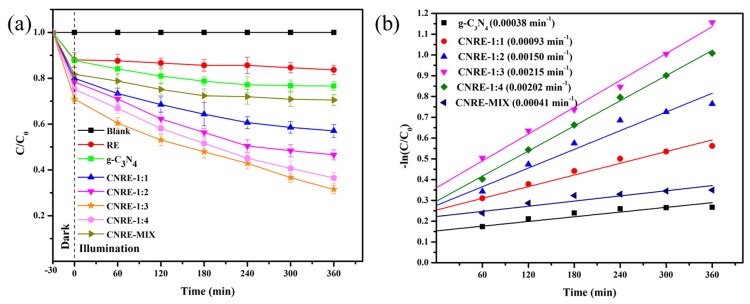
(**a**) Photodegradation of CIP by rectorite, CNRE-1:1, CNRE-1:2, CNRE-1:3, CNRE-1:4, CNRE-MIX and g-C_3_N_4_ under visible light; (**b**) the corresponding first-order kinetics plots.

In this work, CIP was employed as the target pollutant to determine the photoactivity of samples. The photocatalytic activities of rectorite, g-C_3_N_4_, CNRE-1:1_,_ CNRE-1:2, CNRE-1:3, CNRE-1:4 and CNRE-MIX were revealed in Figure 6. As described in Figure 6a, interestingly, the removal rate of CIP was only about 12.3% after adsorption by pure g-C_3_N_4_, which was mainly because of its agglomeration of lamellar nanosheets. As the amount of dicyandiamide increased, the adsorption rate of g-C_3_N_4_/rectorite raised gradually and then decreased. CNRE-1:3 possessed the best adsorption activity and the removal rate of CIP increased to 29.2%. This phenomenon could be attributed to the higher adsorption ability of rectorite as catalyst carrier. In addition, the introduction of rectorite would be beneficial for exfoliation of the stack g-C_3_N_4_, which also greatly promoted its adsorption ability towards CIP. After 6 h illumination, it is apparent that in the presence of rectorite, only about 16.3% removal rate for CIP can be obtained because of adsorption. For pure g-C_3_N_4_, this value reached about 33.4% ascribed to the photocatalysis of g-C_3_N_4_. By contrast, CNRE-1:3 revealed the highest degradation efficiency for CIP (around 70%). Furthermore, the photocatalytic degradation obeyed the first-order kinetics. The kinetics could be denoted as below [15]:

−ln(C_0_/C) = kt (2)

Based on the first-order kinetics, the rate constants of CNRE-1:1, CNRE-1:2, CNRE-1:3, CNRE-1:4, CNRE-MIX and g-C_3_N_4_ were calculated to be 0.00093 min^−1^, 0.00150 min^−1^, 0.00215 min^−1^, 0.00202 min^−1^, 0.00041 min^−1^ and 0.00038 min^−1^, respectively. It is clear that the rate constant of CNRE-1:3 was 5.66 times that of single g-C_3_N_4_ and 5.24 times that of CNRE-MIX. The introduction of natural rectorite not only increased the degradation efficiency but also reduced the usage of g-C_3_N_4_ photocatalyst in practical application. Hence, considering the low cost and abundance of natural rectorite, the as-prepared g-C_3_N_4_/rectorite composite should be an environmentally and economically photocatalytic material in the field of wastewater treatment.

### 3.7. UV-Vis Diffuse Reflection Spectrum

**Figure 7:**
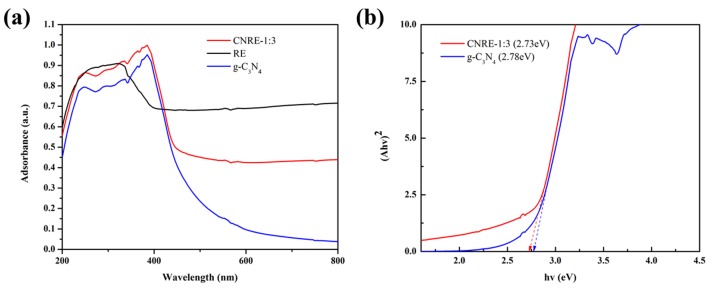
(**a**) UV-vis spectra; (**b**) band gaps of rectorite, g-C_3_N_4_ and CNRE-1:3.

UV-vis diffuse reflectance spectra of rectorite, g-C_3_N_4_ and CNRE-1:3 were depicted in Figure 7a. The intensity of optical absorption of rectorite remained higher, which might be due to that the gray rectorite is easier to absorb light. Besides, the bare g-C_3_N_4_ revealed the absorption edge at 441 nm, which was in good agreement with the observation of previous literature [33]. Furthermore, the band gap of semiconductor can be determined by the formula α_v_ = A(h_v_ − Eg)^n/2^ [34], which revealed the band gap of roughly 2.78 eV and 2.73 eV for g-C_3_N_4_ and CNRE-1:3, respectively. Compared with single g-C_3_N_4_, no obvious enhancement of the absorbance intensity of CNRE-1:3 in visible light region was observed. This should be because the carrier rectorite has no visible light absorption ability. However, the change of optical properties further verified the effective immobilization of g-C_3_N_4_ on rectorite. Hence, we can conclude that the performance improvement mechanisms should be the improvement of adsorption capacity towards CIP, the distribution of catalyst and the effective separation of photogenerated electron-hole pairs, rather than extended light harvesting.

### 3.8. The Reusability and Stability of Catalyst

**Figure 8:**
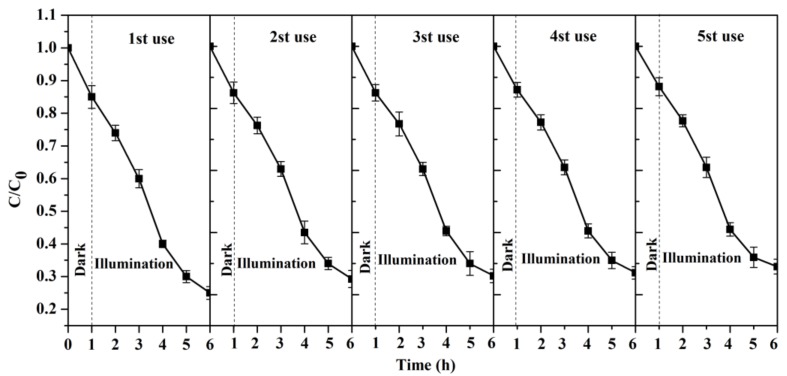
The reusability and stability test of CNRE-1:3.

In order to further study the reusability and stability of CNRE-1:3, five successive photocatalytic experiments were implemented under the same conditions. However, the initial concentration of CIP remained constant in each cycle. The results were given in Figure 8. As the reuse time increases, the photocatalytic degradation rate showed a slight downward trend, since the degradation intermediates of CIP absorbed on the surface of catalyst, resulting in the decrease of photoelectron transfer [35]. However, compared with initial degradation performance, the photocatalytic activity of CNRE-1:3 still maintains a high level after five successive runs. This means that the CNRE-1:3 has great application potential in wastewater treatment with good reusability and low cost.

### 3.9. Proposed CIP Degradation Pathways

**Figure 9:**
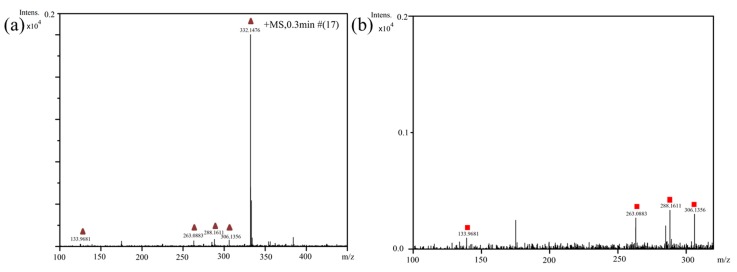
(**a**) Representative mass spectra of CIP; (**b**) magnified mass spectra from 0 to 0.2 of intensity.

**Figure 10:**
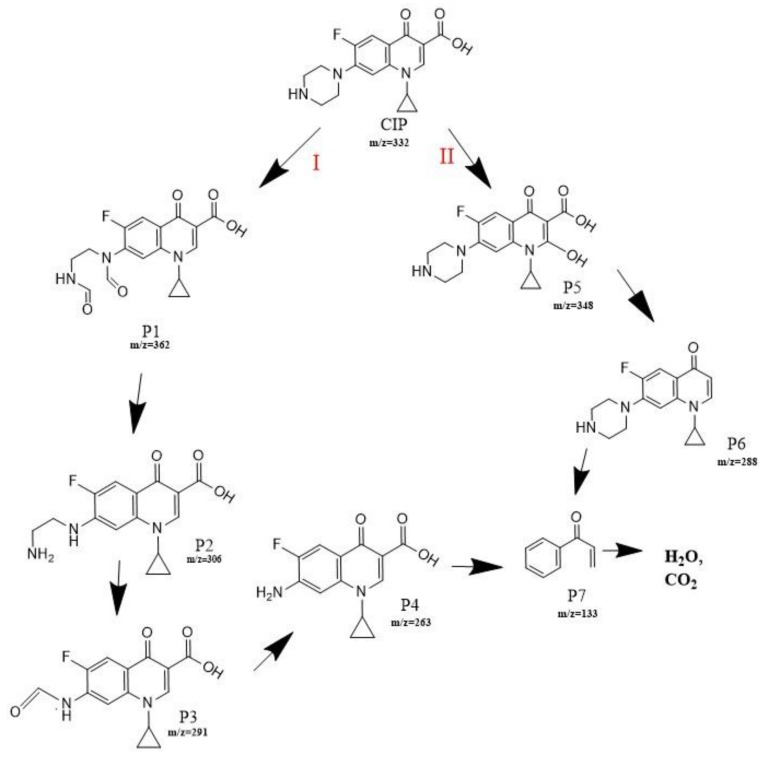
Proposed degradation pathways of CIP by CNRE-1:3 under visible light irradiation.

To better understand the degradation processes of CIP by composite, the intermediate products in catalytic reaction process were analyzed and briefly described in Table 2. The intermediates namely P1 (*m*/*z* = 362), P2 (*m*/*z* = 306), P3 (*m*/*z* = 291), P4 (*m*/*z* = 263), P5 (*m*/*z* = 348), P6 (*m*/*z* = 288) and P7 (*m*/*z* = 133) were reviewed. A representative mass spectra of CIP after 6 h of irradiation was shown in Figure 9. The proposed degradation pathways of CIP by CNRE-1:3 under visible light were revealed in Figure 10. As described in Figure 10, two main decay pathways were presented. The molecular weight of intermediates were apparently decreased in all the pathway, indicating that CIP was decomposed effectively. In pathway I, the piperazine ring was oxidized causing the ring to open. Then, two –CO were lost when P1 transformed into P2 [36,37]. Consulting previous studies [38,39], P3 generated through the processes of hydroxylation and the removal of CHNH_2_ from P2. In the following step, the P4 was generated and the –CO was eliminated from the piperazine ring [40]. The growth of a hydroxyl group should be the possible degradation pathway II. One more hydroxyl group was added to generate P5. P5 turn into P6 because losing water and carboxylic group [41]. Finally, original CIP molecules and intermediate were transformed into P7 and further oxidize to CO_2_ and H_2_O molecular.

### 3.10. Photocatalytic Mechanism

**Figure 11:**
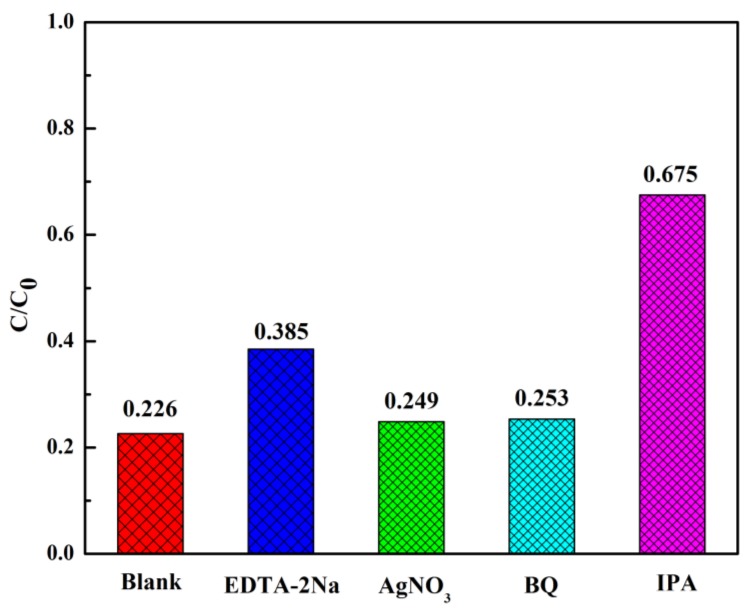
The different effects of active species during photodegradation of CIP.

**Figure 12:**
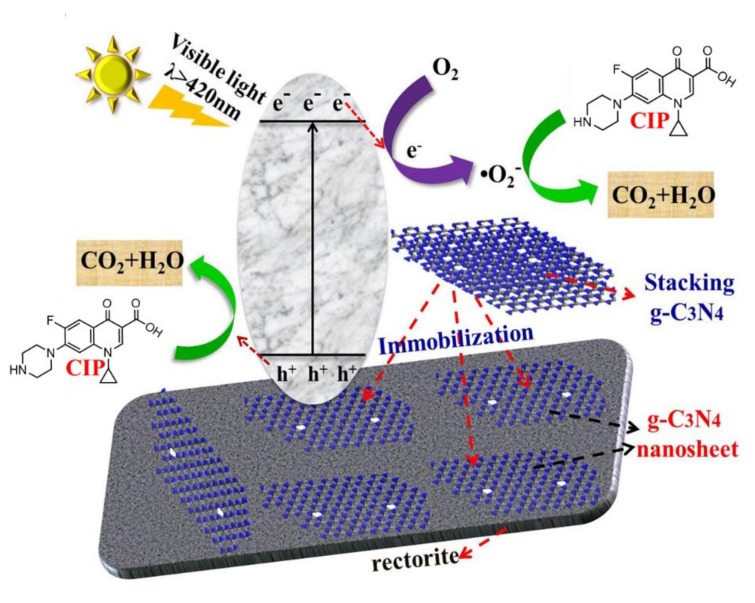
Proposed enhancement mechanisms of g-C_3_N_4_/rectorite composite under visible light.

Since the photocatalytic reaction is tightly related to active species, the free radical experiments has been used to further investigate the degradation process and study the photocatalytic mechanisms. The experimental processes were the same as the photocatalytic activity tests except for adding 10 mM of silver nitrate (AgNO_3_), edentate disodium (EDTA-2Na), isopropanol (IPA) and 1, 4-benzoquinone (BQ). EDTA-2Na can be used to scavenge h^+^, AgNO_3_ was regarded as the scavenger of e^−^ and BQ was applied to scavenge •O_2_^−^ and IPA as •OH scavenger. According to Figure 11, when the BQ and AgNO_3_ were added, the degradation efficiency was barely depressed, demonstrating •O_2_^−^ and e-radicals were not the dominant reactive species in the photodegradation process. Conversely, the trapping of •OH by IPA inhibited the decomposition of CIP significantly and slight decrease also observed in EDTA (hole scavenger). The results suggested that the degradation should be mainly mediated by hydroxyl radicals and holes. For bare g-C_3_N_4_, the frequent recombination of photo-generated electrons and holes result in a lower activity. The probable photocatalytic mechanism for CNRE-1:3 was depicted in Figure 12. After the immobilization of stacking g-C_3_N_4_ on rectorite, the adsorption capacity towards CIP of catalyst would be improved. On the other hand, the introduction of rectorite significantly improved the distribution of the g-C_3_N_4_ nanosheets, which would supply more reactive sites for photodegradation. Furthermore, the formation of the g-C_3_N_4_/rectorite composite could effectively promote the effective separation of photogenerated electron-hole pairs in the photocatalytic process.

## 4. Conclusions

In conclusion, a novel g-C_3_N_4_/rectorite composite was successfully prepared by a facile two-step process. The g-C_3_N_4_ was successfully immobilized and uniformly distributed on layered rectorite, generating a novel “2D/2D” structure. Because of the formation of the “2D/2D” structure, the reactive sites for degrading pollutant were increased. Compared with pure g-C_3_N_4_, the composite revealed higher photocatalytic activities and lower recombination of the photogenerated carrier. During the photocatalytic degradation process, it is evidential that the •OH radicals had the most important effect on the reaction. The reaction rate constant of the prepared g-C_3_N_4_/rectorite composite is 5.66 times that of bare g-C_3_N_4_. Based on the structural characterization analysis, the tight interface contact between g-C_3_N_4_ and rectorite was favorable for decreasing the recombination of e^-^-h^+^ pairs, enhancing the adsorption capacity and photodegradation performance toward the pollutant. The received g-C_3_N_4_/rectorite composite demonstrate a great potential as a cost-effective photocatalyst for the degradation of personal care products and residuals of medicine in wastewater treatment.

## Figures and Tables

**Table 1:** N_2_ adsorption-desorption characteristics of the rectorite, g-C_3_N_4_ and CNRE-1:3 composite.

Sample	${\bar{S}}_{\mathrm{BET}}$ (m^2^/g)	Standard Deviations of S_BET_	$$\bar{P}$$ ore Volume (cm^3^/g)
Rectorite(RE)	28.9	1.3	0.060
g-C_3_N_4_	16.2	2.2	0.046
CNRE-1:3	27.1	1.5	0.062

**Table 2:** Oxidation products of CIP degradation by CNRE-1:3 under visible light irradiation.

Products	Molecular Formula	Structural Formula	*m*/*z*	Name
CIP	C_17_H_18_FN_3_O_3_	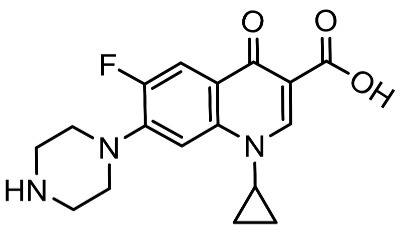	332	ciprofloxacin
P1	C_17_H_16_FN_3_O_5_	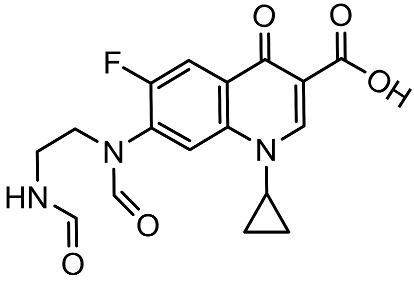	362	1-cyclopropyl-6-fluoro-7-(*N*-(2-formamidoethyl)formamido)-4-oxo-1,4-dihydroquinoline-3-carboxylic acid
P2	C_15_H_16_N_3_FO_3_	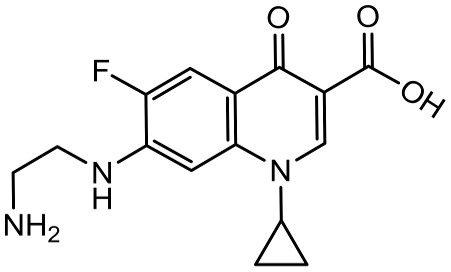	306	7-((2-aminoethyl)amino)-1-cyclopropyl-6-fluoro-4-oxo-1,4-dihydroquinoline-3-carboxylic acid
P3	C_14_H_11_FN_2_O_4_	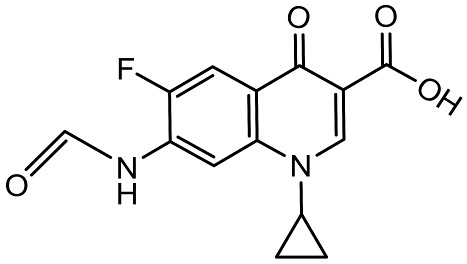	291	1-cyclopropyl-6-fluoro-7-formamido-4-oxo-1,4-dihydroquinoline-3-carboxylic acid
P4	C_13_H_11_FN_2_O_3_	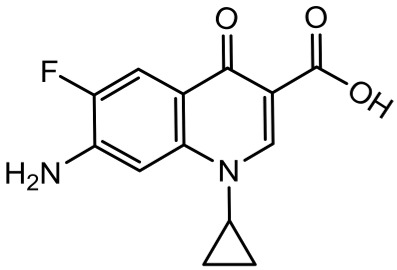	263	7-amino-1-cyclopropyl-6-fluoro-4-oxo-1,4-dihydroquinoline-3-carboxylic acid
P5	C_17_H_18_FN_3_O_4_	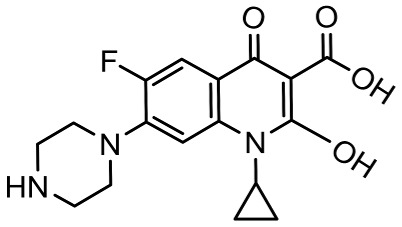	348	1-cyclopropyl-6-fluoro-2-methyl-4-oxo-7-(piperazin-1-yl)-1,4-dihydroquinoline-3-carboxylic acid compound with λ^1^-oxidane (1:1)
P6	C_16_H_18_FN_3_O	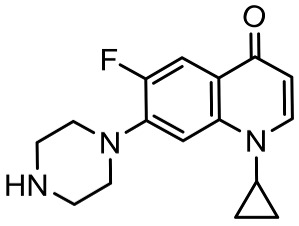	288	1-cyclopropyl-6-fluoro-7-(piperazin-1-yl)quinolin-4(1*H*)-one
P7	C_9_H_8_O	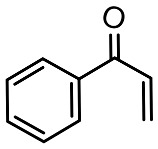	133	1-phenylprop-2-en-1-one
